# SDNN predicts 90-day disability after intravenous thrombolysis: autonomic dysfunction as a novel predictors in acute ischemic stroke

**DOI:** 10.3389/fneur.2026.1756026

**Published:** 2026-02-12

**Authors:** Xiaoyan Wu, Jianping Wang, Liumeng Jian, Li Shi, Jie Li, Jianqiang Zhong, Guoyou Peng, Jianjun Guo

**Affiliations:** Department of Neurology, The Fourth Affiliated Hospital of Guangzhou Medical University, Guangzhou, China

**Keywords:** acute ischemic stroke, autonomic function, autonomic nervous system, heart rate variability, SDNN

## Abstract

**Background:**

Recent studies have clarified the relationship between autonomic dysfunction and ischemic stroke location, etiology, and neurological outcomes. However, few studies have evaluated autonomic nervous system (ANS) function via heart rate variability (HRV) in patients undergoing intravenous thrombolysis. Moreover no HRV parameter has been conclusively established as an independent predictor of unfavorable prognosis in this clinical population.

**Methods:**

We retrospectively analyzed data from acute ischemic stroke (AIS) patients treated with intravenous thrombolysis (IVT) between January 2021 and December 2023. HRV measurements were obtained within 7 days post-stroke to evaluate ANS function. Of the 150 patients included, a unfavorable outcome was defined as a modified Rankin Scale score >2 at 90-days. Multivariate logistic regression adjusted for potential confounders, was used to evaluate associations between HRV parameters and functional outcomes.

**Results:**

Linear regression analyses revealed consistent associations between favorable functional outcomes and HRV parameters reflectiving both sympathetic and parasympathetic activity, assessed at 7- and 90-days post-stroke (all *p* < 0.05). In multivariable logistic regression model, a lower standard deviation of normal-to-normal intervals was identified as an independent Influencing factor of worse modified Rankin Scale scores after adjustment for potential confounders.

**Conclusion:**

Impaired autonomic nervous system function in the acute phase of ischemic stroke may exert a sustained influence on recovery, extending to 90 days post-onset. Standard deviation of normal-to-normal intervals emerged as an independent risk factor for unfavorable prognosis in acute ischemic stroke patients treated with intravenous thrombolysis.

## 1 Introduction

Stroke continues to be a significant global public-health challenge and presents a severe threat to human health. Based on disability—adjusted life years (DALYs), it remains the second leading cause of death globally and the third leading cause of combined mortality and disability (1). In China, ischemic stroke is the predominant subtype, representing 65.3% of all stroke cases (2). Intravenous thrombolysis (IVT), as a standard treatment for acute ischemic stroke (AIS), has been shown to effectively reduce stroke-related disability by facilitating vascular recanalization (3, 4). Although intravenous IVT is a well-established treatment for AIS, post-thrombolysis neurological deterioration remains a substantial clinical issue in a considerable number of patients (5). Therefore, identifying and intervening factors that can affect the efficacy of IVT and is crucial for improving patient outcomes. Currently, most predictive markers focus on the hyperacute phase or short-term outcomes. There is still a gap in identifying transversal prognostic markers that persist from the acute phase into later recovery and can help explain functional outcomes.

A profound pathophysiological association exists between the dysfunction of the autonomic nervous system (ANS) and ischemic stroke. The regulation of the ANS in the brain is not achieved by a single “center” but relies on a widely distributed “central autonomic neural network”. This network primarily encompasses the insula, anterior cingulate cortex, prefrontal cortex, amygdala, hypothalamus, and brain stem (6). These regions collaborate through intricate connections to implement “top–down” regulation of the heart. When a stroke damages these network nodes or disrupts their connectivity, it leads to ANS abnormalities. There is evidence suggesting that there may be right - sided dominance of neural structures that control heart rate and heart rate variability (HRV) (7). This observation elucidates the pronounced disruption of HRV associated with damage to the right hemisphere, particularly regions integral to the limbic system and the salience network. Emerging evidence further indicates that post-stroke alterations in HRV may stem from impaired functional connectivity within resting-state brain networks (8).

While the pathophysiological mechanisms of the autonomic nervous system (ANS) in ischemic stroke are well-characterized, its clinical utility warrants further investigation. HRV, a non-invasive and quantifiable biomarker of ANS function, demonstrates significant clinical potential in this context. It provides an objective measure of the often-underestimated autonomic dysfunction following stroke: diminished HRV correlates with more severe neurological impairment, as indicated by higher NIHSS scores, and reflects both injury to autonomic regulatory centers and the magnitude of systemic stress mediated by the brain-heart axis (9). Furthermore, dynamic alterations in HRV serve as a robust predictor for the risk of major post-stroke complications, including malignant arrhythmias, cardiac injury, and infections (10), thereby providing a powerful tool for risk stratification in the acute phase—for instance, by identifying high-risk patients during the hyperacute stage. Additionally, the recovery trajectory of HRV demonstrates a significant association with long-term functional outcomes, maintaining substantial prognostic value even throughout the chronic rehabilitation period (11). In summary, the integration of multidimensional information-encompassing stroke severity, complication risk, and neural repair potential-enables HRV to serve as an objective and multifaceted quantitative tool across the full clinical management continuum, from acute-phase early warning to long-term outcome evaluation. Consequently, HRV has emerged not merely as an indicator of autonomic function, but as an independent prognostic biomarker that reflects the integrative capacity of higher-order neural networks, with direct implications for adverse neurological outcomes and elevated complication risk.

Building upon this foundation, the present study not only seeks to corroborate the association between HRV and stroke but places greater emphasis on its translation into a clinical tool possessing distinct and independent prognostic value for IVT-treated patients. We further introduce the novel concept that autonomic function exerts a continuous influence across the entire continuum of stroke recovery. This perspective offers a new theoretical framework and an evaluative target for subsequent investigations into neuroprotective or neuromodulatory interventions.

## 2 Materials and methods

### 2.1 Study population

**Figure 1:**
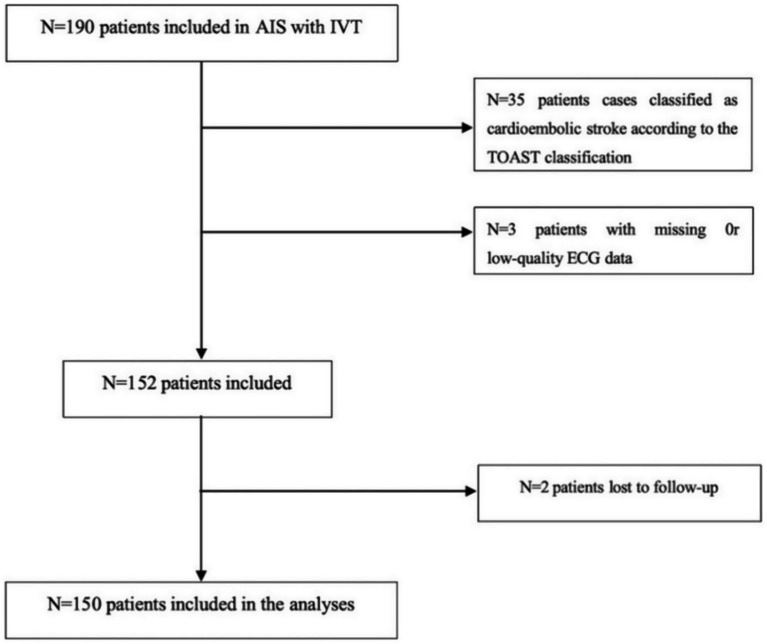
Algorithm for inclusion/exclusion of patients.

This retrospective analysis included patients with AIS who were admitted to the Department of Neurology at The Fourth Affiliated Hospital of Guangzhou Medical University from January 2021 to December 2023. Eligible participants were those who received IVT within 4.5 h of symptom onset. Inclusion criteria comprised: (1) performance of 24-h Holter monitoring within 24 h post-thrombolysis and within 7 days of symptom onset; and (2) fulfillment of IVT eligibility according to the 2018 Chinese AIS Guidelines (11). Exclusion criteria were: (1) contraindications to IVT as specified in the aforementioned guidelines; (2) pre-existing cardiac disorders known to influence heart rate variability (HRV) parameters, including dilated cardiomyopathy, hypertrophic cardiomyopathy, acute coronary syndrome, heart failure, arrhythmias, or atrial fibrillation; (3) a medical history of hyperthyroidism, epilepsy, Parkinson’s disease, multiple sclerosis, anemia, or other conditions potentially affecting HRV; and (4) use of medications impacting the cardiac autonomic nervous system (e.g., digoxin, levodopa, *β*-blockers) prior to admission; (5) exclusion of participants from whom reliable HRV analysis could not be derived from 24-h electrocardiogram recordings owing to substantial signal interference; (6) presence of unstable clinical conditions, such as systemic inflammatory states, renal or hepatic failure, intracranial neoplasms, or active infectious diseases; (7) exclusion of cases identified as cardioembolic stroke based on the TOAST classification, to ensure that the observed HRV alterations more accurately reflect the specific impact of the acute stroke event on autonomic nervous system function. Written informed consent was obtained from all participants or their legally authorized representatives. The study protocol received approval from the Ethics Committee of The Fourth Affiliated Hospital of Guangzhou Medical University, and all procedures were conducted in accordance with the principles of the World Medical Association Declaration of Helsinki. The research flowchart is shown in Figure 1.

### 2.2 Clinical assessments

All patients were administered recombinant tissue plasminogen activator (rt-PA; alteplase, Boehringer Ingelheim, batch 202010) at a dose of 0.9 mg/kg (maximum 90 mg). Clinical evaluations were performed within 24 h of hospital admission. Collected data encompassed demographic characteristics, vascular risk factors (including hypertension, diabetes, smoking history, prior stroke, coronary artery disease, and medication use), NIHSS scores before and after thrombolysis, and stroke subtypes classified according to the TOAST criteria. Functional outcomes were assessed using the modified Rankin Scale (mRS) at 90 days, with a score >2 defined as an unfavorable outcome. Currently, there is no universally accepted definition for DNI. In the present study, favorable outcomes at 7 days post-treatment were defined as follows (12, 13): (1) an improvement of ≥4 points in NIHSS score from baseline; or (2) an improvement of ≥20% in NIHSS score from baseline. The NIHSS was assessed at admission in the emergency department by a certified neurologist prior to thrombolysis. At 90 days post-stroke, the mRS score was obtained via a standardized telephone interview conducted by a trained research coordinator, who was blinded to all baseline clinical information and HRV data.

### 2.3 Heart rate variability

HRV parameters were derived from 24-h Holter monitoring (B19900, Shenzhen Boying Dynamic ECG Analysis System), conducted within a post-onset window of 24 h to 7 days. Standard 12-lead ECG signals (I, II, III, aVF, aVL, aVR, V1–V6) were acquired to enable comprehensive cardiac cycle analysis. All assessments were carried out in a quiet examination room. To reduce potential confounding effects of circadian rhythm on heart rate variability, measurements were scheduled between 9:00 a.m. and 10:00 a.m., with ambient temperature maintained at 20 °C–24 °C. Prior to testing, patients rested in a supine position for 10 min. Beat-to-beat recordings were analyzed using specialized software to generate cardiac cycle patterns. For the purposes of this study, the wake phase was defined as 08:00 to 22:00, and the sleep phase as 22:00 to 08:00 the following day.

ECG monitoring was performed under naturalistic conditions (encompassing both active and resting states) at a sampling frequency of 250 Hz. Heart rate variability (HRV) was assessed across two primary domains: time-domain indices, which comprised (1) the standard deviation of normal-to-normal R-R intervals (SDNN), reflecting the overall variability in sinus rhythm, and (2) the root mean square of successive differences between adjacent R-R intervals (RMSSD), representing beat-to-beat variance in heart rate. The frequency-domain parameters assessed were: (1) Low Frequency (LF), corresponding to the spectral power between 0.04 and 0.15 Hz; (2) High Frequency (HF), representing the spectral power from 0.15 to 0.40 Hz; (3) Very Low Frequency (VLF), denoting the spectral power below 0.04 Hz; and (4) the ratio of low-frequency to high-frequency power (LF/HF). Additionally, a geometric approach based on R-R interval histogram distributions was applied-specifically, the Heart Rate Variability Triangular Index. This index is derived as the quotient of the total number of normal R-R intervals divided by the maximum bin height of the histogram, providing a geometric assessment of overall heart rate variability.

### 2.4 Statistical analysis

All statistical analyses were conducted using SPSS (version 26.0) and R (version 4.4.1). Normality of variable distributions was evaluated with the Kolmogorov–Smirnov test. Normally distributed continuous variables are presented as mean ± standard deviation and compared between independent groups using Student’s *t*-test. Non-normally distributed continuous variables are summarized as median (interquartile range) and compared using the Mann–Whitney U test. Categorical variables are reported as frequencies and percentages, with between-group differences assessed using the chi-square test or Fisher’s exact test, as appropriate.

To assess the association between HRV and 7-day or 3-month clinical outcomes in patients receiving IVT, multivariable logistic regression models were employed. Sensitivity analyses were conducted using four distinct models: (1) unadjusted; (2) adjusted for age and sex; (3) additionally adjusted for vascular risk factors (including smoking, alcohol consumption, hypertension, diabetes, dyslipidemia, and prior ischemic stroke); and (4) further adjusted for clinical parameters (including admission systolic and diastolic blood pressure, heart rate, fasting glucose, NIHSS score, onset-to-admission time, TOAST classification, and antihypertensive medication). All statistical tests were two-sided, with significance defined as *p* < 0.05.

## 3 Result

### 3.1 Baseline characteristics of AIS patients treated with intravenous alteplase

At the 7-day follow-up, unfavorable outcomes were recorded in 43 patients (28.7%), a proportion that decreased to 40 patients (26.7%) at 90 days. Baseline characteristics, stratified according to 90-day clinical outcomes, are summarized in Table 1. Patients with unfavorable outcomes were significantly older than those with favorable outcomes (mean age 69.33 vs. 62.68 years). A history of ischemic stroke was significantly more prevalent in the unfavorable outcome group (35.0% vs. 14.7%; *p* = 0.002). Several key clinical parameters-including fasting glucose (6.02 vs. 5.19 mmol/L, *p* = 0.043), systolic blood pressure (162.15 vs. 153.04 mmHg, *p* = 0.038), and admission NIHSS scores (median 9 vs. 4; *p* < 0.001)—differed significantly between groups, with more favorable values observed among patients with positive outcomes. In addition, the distribution of TOAST etiological subtypes differed significantly between the two outcome groups (*p* = 0.001).

**Table 1:** Baseline characteristics of AIS patients treated with intravenous alteplase.

Variables	Unfavorable outcome	Favorable outcomes	*χ*^2^/t/z	*p value
	*N* = 40	*N* = 110		
Demographics				
Sex, men, *n* (%)	33 (82.5%)	82 (74.5%)	1.038	0.308
Age, y	69.33 ± 10.60	62.68 ± 10.79	3.35	0.001
Vascular risk factors				
Cigarette smoking, *n* (%)	9 (22.5%)	26 (23.6%)	0.021	0.884
Hypertension, *n* (%)	33 (82.5%)	91 (82.7%)	0.001	0.974
Previous ischemic stroken, *n* (%)	14 (35.0%)	14 (12.7%)	9.585	0.002
Diabetes, *n* (%)	15 (37.5%)	44 (40.0%)	0.077	0.782
Previous history of medication use				
Anti-platelet agents, *n* (%)	7 (17.5%)	12 (10.9%)	1.152	0.283
Anti-hypertension agents, *n* (%)	22 (55.0%)	49 (44.5%)	1.286	0.257
Hypoglycemic agent, *n* (%)	9 (22.5%)	28 (25.4%)	0.138	0.710
Antihyperlipidemic agents, *n* (%)	7 (17.5%)	13 (11.8%)	0.819	0.365
Clinical data				
SBP, mm Hg	162.15 ± 23.85	153.04 ± 23.41	2.098	0.038
DBP, mm Hg	87.78 ± 12.12	88.50 ± 13.04	−0.307	0.760
Serum fasting glucose, mmol/L	6.02 (4.84, 7.56)	5.19 (4.5, 6.61)	−2.027	0.043
Admission NIHSS score	9.00 (6.00, 16.00)	4.00 (3.00, 6.00)	−4.984	<0.001
TOAST			13.883	0.001
LAA	26 (65.0%)	44 (40.0%)		
SAO	8 (20.0%)	59 (53.6%)		
ODC or undetermined cause	6 (15.0%)	7 (6.4%)		
Laboratory indicators				
CHOL, mmol / L	5.06 ± 1.27	5.05 ± 1.32	0.04	0.968
LDL, mmol / L	3.03 ± 0.96	3.11 ± 0.99	−0.452	0.652
Uric acid, μmmol/L	362.00 (291.5, 436.25)	357.00 (290.50, 421.75)	−0.431	0.666
FIB, g/L	3.31 (2.62, 3.95)	3.03 (2.57, 3.53)	−1.481	0.139

Age, DBP, SBP, CHOL, and LDL are expressed as mean ± SD and were analyzed using the Student t test. Serum fasting glucose, uric acid, FIB, and onset to admission time are expressed as median (interquartile range) and were compared using the Mann–Whitney U test. The other variables are expressed as *n* (%) and were analyzed using the *χ*^2^ test or Fisher exact test. CHOL, total cholesterol; DBP, diastolic blood pressure; FIB, fibrinogen; LDL stands for low density lipoprotein cholesterol; NIHSS, National Institutes of Health Stroke Scale; SBP, systolic blood pressure.

### 3.2 The association between heart rate variability parameters and 90-day unfavorable outcome in patients with intravenous thrombolysis

Table 2 compares heart rate variability (HRV) parameters between patients with favorable and unfavorable 90-day functional outcomes. Individuals in the favorable outcome group exhibited significantly higher values across both time-domain and frequency-domain measures. In the time domain, SDNN—an indicator of overall autonomic variability-was elevated in the favorable outcome group (101.71 ± 33.74 ms vs. 79.72 ± 19.93 ms), indicating enhanced global autonomic regulatory capacity. Similarly, RMSSD, which reflects vagally-mediated heart rate modulation, was also significantly greater in this group [median: 25.89 ms (IQR: 20.57–33.40) vs. 20.06 ms (16.22–28.43)]. Furthermore, the HRV triangular index, a geometric measure of overall heart rate variability, was also elevated in the favorable outcome group [16.5 (12.0–20.25) vs. 12.5 (10.25–16.0)]. In the frequency domain, a consistent pattern was evident across all spectral bands. High-frequency (HF) power, an indicator of vagal activity, was nearly twofold higher in the favorable outcome group [140.46 (74.98–231.44) ms^2^ vs. 82.1 (34.68–163.59) ms^2^]. Similarly, low-frequency (LF) power, which is linked to baroreflex function and more complex neurohumoral regulation, was significantly greater [259.91 (152.79–482.02) ms^2^vs. 146.81 (62.61–319.09) ms^2^]. Very-low-frequency (VLF) power, reflecting ultra-slow cyclic variations, was also markedly increased in the favorable outcome group [995.46 (626.24–1541.90) ms^2^ vs. 529.12 (285.36–1134.42) ms^2^]. In contrast, the LF/HF ratio did not exhibit a significant difference between groups. Owing to persistent controversy surrounding the interpretation of the LF/HF ratio as a direct marker of sympathovagal balance, its lack of statistical significance in the present analysis does not substantively impact the overall conclusions of the study.

**Table 2:** The association between heart rate variability parameters and 90-day unfavorable outcome in patients with intravenous thrombolysis.

HRV	Unfavorable outcome	Favorable outcome	*t*	^*^ *p*
	*N* = 40	*N* = 110		
SDNN	79.72 ± 19.93	101.71 ± 33.74	−3.878	<0.001
Awake SDNN	67.98 ± 17.18	87.25 ± 28.43	−4.023	<0.001
Asleep SDNN	76.96 ± 26.05	97.79 ± 36.51	−3.312	0.001
RMSSD	20.06 (16.22, 28.43)	25.89 (20.57, 33.4)	−2.89	0.004
Awake RMSSD	19.69 (16.36, 25.91)	24.49 (19.24, 31.24)	−2.779	0.005
Asleep RMSSD	21.12 (16.47, 29.69)	27.3 (19.61, 36.41)	−2.663	0.008
HF	82.1 (34.68, 163.59)	140.46 (74.98, 231.44)	−2.856	0.004
Awake HF	73.28 (29.15, 151.38)	121.37 (56.54, 220.89)	−2.55	0.011
Asleep HF	91.59 (39.74, 187.72)	156.91 (82.09, 319.65)	−2.992	0.003
LF	146.81 (62.61, 319.09)	259.91 (152.79, 482.02)	−3.289	0.001
Awake LF	126.01 (56.13, 271.36)	238.5 (143.34, 448.86)	−3.464	0.001
Asleep LF	183.79 (72.42, 325.79)	287.52 (155.23, 528.38)	−3.009	0.003
VLF	529.12 (285.36, 1134.42)	995.46 (626.24, 1541.9)	−3.396	0.001
Awake VLF	513.31 (223.07, 1058.71)	885.16 (552.26, 1402.99)	−3.328	0.001
Asleep VLF	653.66 (366.88, 1465.33)	1171.22 (752.73, 1684.96)	−3.149	0.002
HRV Triangular Index	12.5 (10.25, 16)	16.5 (12, 20.25)	−2.91	0.004
LF/HF ratio	1.99 (1.12, 3.04)	1.91 (1.24, 2.77)	−0.223	0.823

HF, high frequency; HRV, heart rate variability; LF, low frequency; VLF, very low frequency; RMSSD, square root of the mean of the sum of the squares of differences between adjacent normal-to-normal intervals; SDNN, standard deviation of N–N intervals.

### 3.3 The association between heart rate variability parameters and 90-day unfavorable outcome in patients with intravenous thrombolysis

Given the potential confounding effects of demographic characteristics and vascular risk factors on heart rate variability and autonomic nervous system function, we employed a multiple linear regression model to adjust for key covariates, thereby allowing a more accurate assessment of the independent association between HRV parameters and clinical outcomes. The adjusted analysis revealed that SDNN remained independently associated with patients’ 90-day functional outcome (Table 3). This finding suggests that, even after accounting for common confounders, the autonomic functional status reflected by early post-stroke SDNN remains a valid predictor of 90-day clinical prognosis. Therefore, post-stroke HRV and autonomic function may not only serve as a reference for prognostic evaluation but could also represent a potential target for future interventions aimed at improving patients’ long-term functional recovery.

**Table 3:** The association between heart rate variability parameters and 90-day unfavorable outcome in patients with intravenous thrombolysis.

HRV	Unadjusted		Adjusted age +sex		Adjusted vascular risk^a^		Adjusted stroke data^b^		Adjusted data^c^	
	*β*	^*^p value	*β*	^*^p value	*β*	^*^p value	*β*	^*^p value	*β*	^*^p value
SDNN	0.027	<0.001	0.022	0.004	0.027	0.002	0.029	0.005	0.024	0.010
Awake SDNN	0.035	<0.001	0.029	0.003	0.036	0.001	0.040	0.003	0.031	0.007
Asleep SDNN	0.021	0.002	0.016	0.018	0.019	0.012	0.018	0.034	0.016	0.055
RMSSD	0.033	0.059	0.027	0.088	0.028	0.084	0.037	0.038	0.031	0.076
Awake RMSSD	0.032	0.077	0.029	0.087	0.030	0.080	0.039	0.035	0.033	0.070
Asleep RMSSD	0.030	0.054	0.023	0.110	0.023	0.105	0.033	0.044	0.027	0.090
LF	0.002	0.012	0.002	0.112	0.002	0.125	0.002	0.118	0.002	0.147
Awake LF	0.003	0.007	0.003	0.072	0.002	0.081	0.002	0.094	0.002	0.112
Asleep LF	0.002	0.023	0.001	0.175	0.001	0.167	0.001	0.138	0.001	0.196
HF	0.001	0.238	0.001	0.298	0.001	0.242	0.001	0.117	0.001	0.201
Awake HF	0.001	0.307	0.001	0.329	0.001	0.255	0.001	0.127	0.001	0.218
Asleep HF	0.001	0.157	0.001	0.245	0.001	0.215	0.001	0.097	0.001	0.162
VLF	0.001	0.002	0.001	0.037	0.001	0.047	0.001	0.153	0.001	0.157
Awake VLF	0.001	0.003	0.001	0.045	0.001	0.051	0.001	0.113	0.001	0.137
Asleep VLF	0.001	0.004	0.001	0.046	0.001	0.064	<0.001	0.223	<0.001	0.198
HRV Triangular Index	0.054	0.099	0.029	0.376	0.029	0.369	0.045	0.205	0.034	0.3292
LF/HF	0.020	0.893	−0.060	0.694	−0.075	0.636	−0.263	0.162	−0.177	0.318

a Adjusted for age, sex, and vascular risk factors (including cigarette smoking, hypertension, diabetes, dyslipidemia, and previous ischemic stroke).

b Adjusted for age, sex, vascular risk factors, and clinical data (including admission systolic blood pressure, admission diastolic blood pressure, serum fasting glucose, admission National Institutes of Health Stroke Scale score, and antihypertensive agents).

c Adjusted for age, sex, admission National Institutes of Health Stroke Scale score, admission systolic blood pressure, serum fasting glucose.

### 3.4 The association between heart rate variability parameters and 7-day unfavorable outcome in patients with intravenous thrombolysis

**Figure 2:**
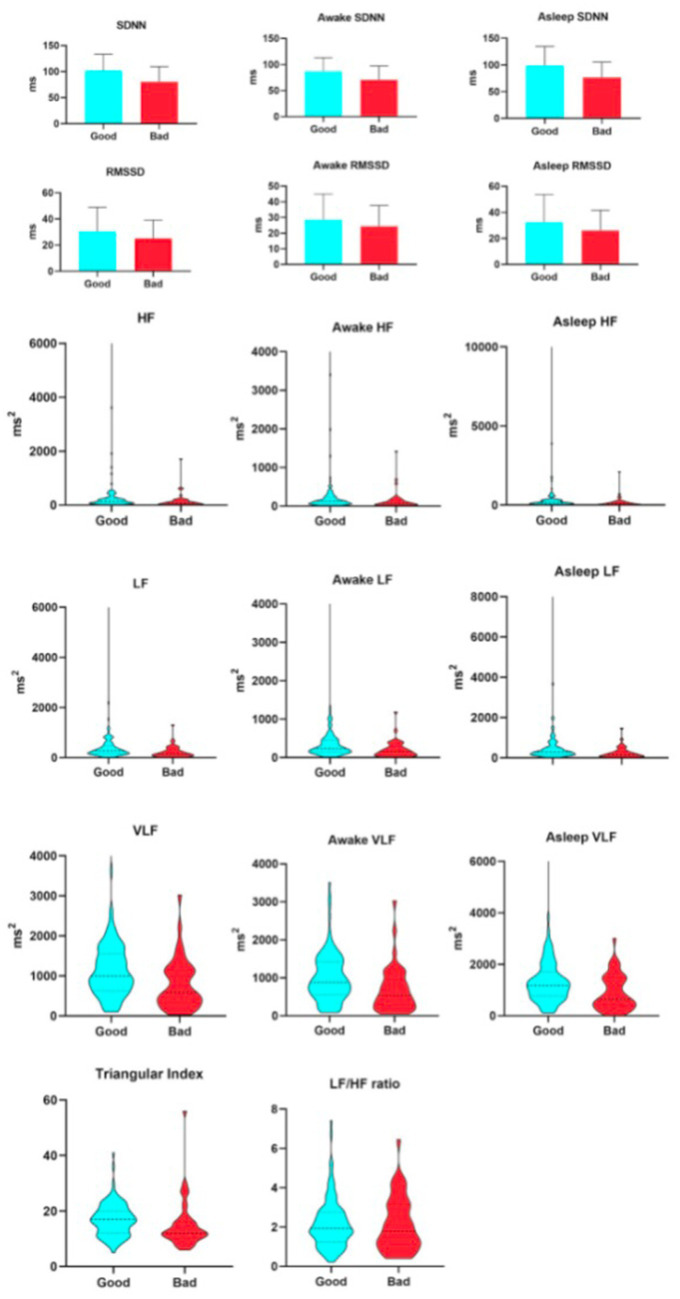
Relationship between heart hate variability parameters and 7-day unfavorable outcome.

**Figure 3:**
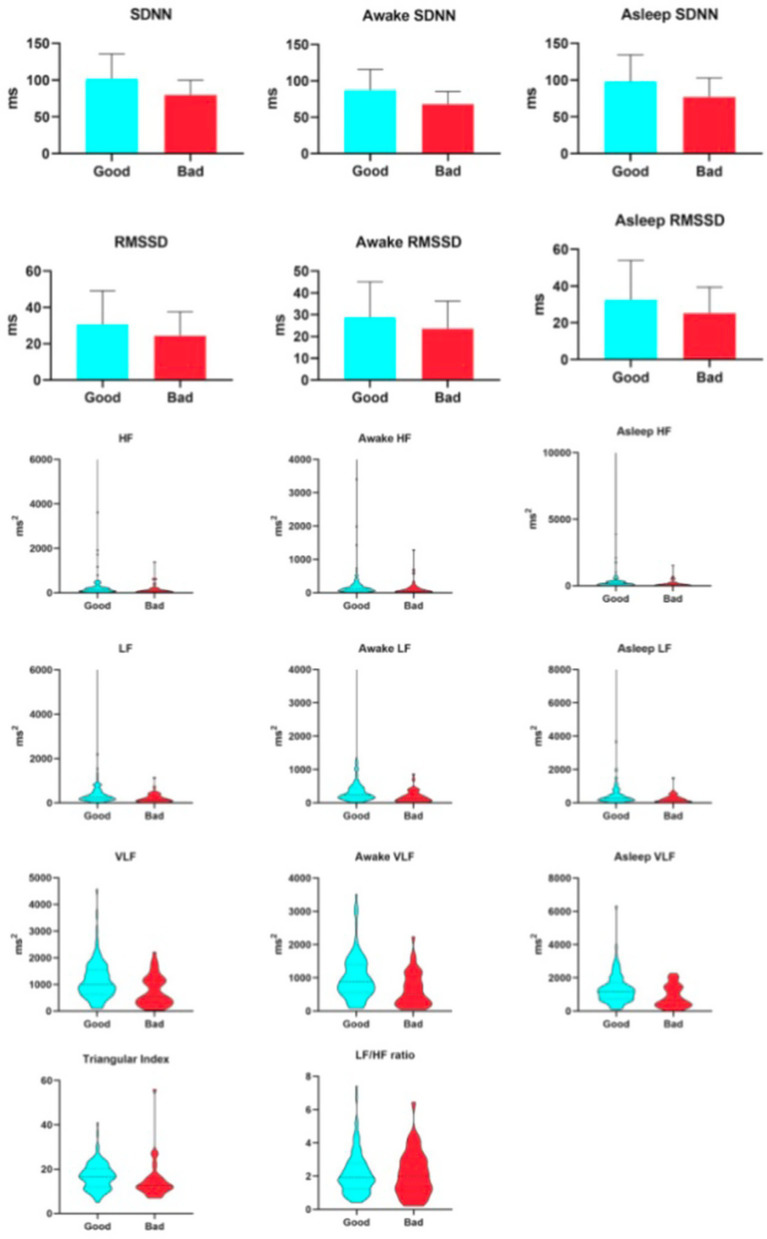
Relationship between heart hate variability parameters and 90-day unfavorable outcome.

As shown in Table 4, when assessed at 7 days after stroke onset, all primary HRV parameters demonstrated significant associations with 90-day clinical outcomes, with generally lower values observed in the unfavorable prognosis group. Specifically, among the time-domain indices, SDNN—reflecting overall autonomic regulatory capacity—was 80.58 ± 4.44 ms in the unfavorable outcome group versus 101.98 ± 3.03 ms in the favorable outcome group. RMSSD, representing rapid vagally-mediated modulation, had a median value of 20.31 ms (IQR: 16.52–29.23 ms) in the unfavorable group and 25.76 ms (20.8–33.39 ms) in the favorable group. The HRV triangular index, which comprehensively evaluates the overall HRV distribution, was 12 (10–15) in the unfavorable group and 17 (12–19) in the favorable group. Regarding frequency-domain parameters, HF, indicative of vagal activity, showed a median of 85.75 ms^2^ (33.69–168.59 ms^2^) in the unfavorable group and 138.89 ms^2^ (76.03–248.28 ms^2^) in the favorable group. LF, related to baroreflex and neurohumoral regulation, was 145.67 ms^2^ (67.21–325.67 ms^2^) in the unfavorable group and 264.42 ms^2^ (154.06–477.09 ms^2^) in the favorable group. VLF, reflecting ultra-slow cyclic activity, was 588.48 ms^2^ (328.17–1130.69 ms^2^) in the unfavorable group and 996.44 ms^2^ (628.05–1556.63 ms^2^) in the favorable group. It is worth reiterating that the LF/HF ratio did not show a statistically significant difference between the two groups. From the initiation of thrombolysis until 7 days post-stroke, patients underwent continuous HRV monitoring. As illustrated in Figure 2, all HRV parameters remained consistently and significantly lower in patients with unfavorable outcomes compared to those with favorable outcomes, both at 7 days and at 90 days after stroke (see Figure 3).

**Table 4:** The association between heart rate variability parameters and 7-day unfavorable outcome in patients with intravenous thrombolysis.

HRV	Unfavorable outcome	Favorable outcome	t/Z	^*^ *p*
	*N* = 43	*N* = 107		
SDNN	80.58 ± 4.44	101.98 ± 3.03	−3.858	<0.001
Awake SDNN	70.43 ± 4.06	86.80 ± 2.53	−3.448	0.001
Asleep SDNN	76.46 ± 4.39	98.57 ± 3.45	−3.618	<0.001
RMSSD	20.31 (16.52, 29.23)	25.76 (20.8, 33.39)	−2.600	0.009
Awake RMSSD	19.65 (16.43, 26.1)	24.66 (19.01, 31.04)	−2.554	0.011
Asleep RMSSD	21.31 (16.42, 30.68)	27.06 (19.63, 36.15)	−2.462	0.014
HF	85.75 (33.69, 168.59)	138.89 (76.03, 248.28)	−2.994	0.003
Awake HF	75.18 (29.06, 156.31)	121.78 (56.66, 226.19)	−2.6	0.009
Asleep HF	95.41 (39.24, 195.73)	155.11 (82.36, 324.24)	−3.165	0.002
LF	145.67 (67.21, 325.67)	264.42 (154.06, 477.09)	−3.385	0.001
Awake LF	154.46 (63.15, 287.66)	237.62 (142.88, 452.91)	−3.339	0.001
Asleep LF	160.44 (72.8, 322.89)	288.15 (158.25, 521.38)	−3.298	0.001
VLF	588.48 (328.17, 1130.69)	996.44 (628.05, 1556.63)	−3.344	0.001
Awake VLF	545.22 (279.68, 965.53)	887.95 (556.4, 1418.15)	−3.227	0.001
Asleep VLF	645.71 (392.97, 1480.19)	1178.03 (774.11, 1712.99)	−3.169	0.002
HRV Triangular Index	12 (10, 16)	17 (12, 20)	−2.923	0.003
LF/HF ratio	1.78 (1.02, 3.14)	1.92 (1.24, 2.76)	−0.434	0.664

HF, high frequency; HRV, heart rate variability; LF, low frequency; VLF, very low frequency; RMSSD, square root of the mean of the sum of the squares of differences between adjacent normal-to-normal intervals; SDNN, standard deviation of N–N intervals.

### 3.5 The association between heart rate variability parameters and 7-day unfavorable outcome in patients with intravenous thrombolysis

As shown in Table 5, after adjusting for key covariates, SDNN remained independently associated with clinical outcomes at 7 days after onset. This finding further supports the role of SDNN as an independent predictor of prognosis in patients after acute stroke and suggests its potential as a biomarker reflecting autonomic nervous system function.

**Table 5:** The association between heart rate variability parameters and 7-day unfavorable outcome in patients with intravenous thrombolysis.

HRV	Unadjusted		Adjusted age +sex		Adjusted vascular risk^a^		Adjusted stroke data^b^		Adjusted data^c^	
	*β*	^*^p value	*β*	^*^p value	*β*	^*^p value	*β*	^*^p value	*β*	^*^p value
SDNN	0.026	<0.001	0.021	0.004	0.028	0.001	0.027	0.004	0.024	0.006
Awake SDNN	0.028	<0.001	0.022	0.011	0.029	0.004	0.026	0.016	0.023	0.023
Asleep SDNN	0.022	0.002	0.018	0.007	0.025	0.002	0.023	0.008	0.020	0.013
RMSSD	0.025	0.099	0.021	0.145	0.025	0.096	0.026	0.088	0.025	0.103
Awake RMSSD	0.024	0.135	0.021	0.156	0.026	0.103	0.026	0.093	0.025	0.108
Asleep RMSSD	0.024	0.090	0.018	0.171	0.021	0.115	0.023	0.098	0.022	0.017
LF	0.002	0.015	0.001	0.127	0.002	0.091	0.001	0.152	0.001	0.145
Awake LF	0.003	0.013	0.002	0.109	0.002	0.084	0.002	0.160	0.002	0.145
Asleep LF	0.002	0.020	0.001	0.150	0.001	0.103	0.001	0.138	0.001	0.152
HF	0.001	0.219	0.001	0.284	0.001	0.225	0.001	0.157	0.001	0.190
Awake HF	0.001	0.274	0.001	0.297	0.001	0.232	0.001	0.157	0.001	0.195
Asleep HF	0.001	0.157	0.001	0.250	0.001	0.201	0.001	0.141	0.001	0.166
VLF	0.050	0.114	0.029	0.349	0.033	0.303	0.036	0.258	0.033	0.282
Awake VLF	0.001	0.004	0.001	0.367	0.001	0.042	0.001	0.171	0.001	0.171
Asleep VLF	0.001	0.009	0.001	0.102	0.001	0.071	0.001	0.205	<0.001	0.221
HRV Triangular Index	0.001	0.004	0.001	0.053	0.001	0.035	<0.001	0.166	<0.001	0.157
LF/HF	0.026	0.854	−0.058	0.696	−0.080	0.609	−0.204	0.267	−0.140	0.401

a Adjusted for age, sex, and vascular risk factors (including cigarette smoking, hypertension, diabetes, dyslipidemia, and previous ischemic stroke).

b Adjusted for age, sex, vascular risk factors, and clinical data (including admission systolic blood pressure, admission diastolic blood pressure, serum fasting glucose, admission National Institutes of Health Stroke Scale score, and antihypertensive agents).

c Adjusted for age, sex, admission National Institutes of Health Stroke Scale score, admission systolic blood pressure, serum fasting glucose.

## 4 Discussion

This observational investigation of AIS patients receiving IVT reveals that autonomic dysfunction constitutes a significant and independent determinant of long-term functional recovery. A single assessment conducted during the acute post-stroke phase (within 7 days) indicated that individuals with unfavorable outcomes already displayed markedly depressed HRV indices. This acute-phase HRV reduction signature was significantly correlated with poor functional status at both short-term (7 days post-thrombolysis) and long-term (90 days post-thrombolysis) follow-ups. Crucially, even after adjustment for established risk factors—including age, stroke history, and baseline metabolic parameters—reduced SDNN persisted as an independent predictor of unfavorable 90-day functional outcome. These observations suggest that post-stroke autonomic dysfunction, representing a distinct pathological mechanism separate from conventional risk factors, exerts a substantial influence on long-term recovery. Consequently, SDNN holds promise as a viable biomarker for stratifying this risk.

The ANS, which is regulated by the dynamic interplay between the sympathetic and parasympathetic branches, coordinates physiological responses to diverse stressors in order to maintain homeostasis (14, 15). HRV has become an established clinical tool for evaluating ANS function and stratifying cardiovascular risk (13). Dysfunction of the ANS under stress, characterized particularly by an imbalance between sympathetic activity and vagal tone—the principal effector of the parasympathetic nervous system—can present as altered HRV. Such HRV alterations are linked to several stroke risk factors, including hypertension and diabetes (16–19). AIS elicits a maladaptive autonomic response that can worsen existing metabolic dysregulation. These stress-induced metabolic alterations subsequently function as secondary stressors, further exacerbating autonomic dysfunction-characterized by heightened sympathetic activity and diminished vagal tone—thereby elevating cerebrovascular and cardiovascular risk. This interaction establishes a self-sustaining vicious cycle that may ultimately compromise clinical outcomes. Given this pathophysiology, HRV, as a biomarker of systemic stress, shows promise for prognostic stratification in patients with cerebrovascular and cardiovascular diseases.

Building on prior evidence that IVT facilitates the assessment of ANS function (20), our study further establishes HRV and autonomic dysfunction as key prognostic risk factors in ischemic stroke patients—an association that persists even among those who received thrombectomy. Previous studies have shown that neurological improvement at 7 days post-thrombolysis independently predicts functional outcome at 90 days (13). The study results show that at both the 7-day and 90-day time points after thrombolysis, HRV parameters were consistently and significantly lower in the unfavorable-outcome group compared to the favorable-outcome group. This indicates a stable association between early autonomic nervous system function—as characterized by HRV—and long-term functional recovery following thrombolysis. Consequently, in hospitalized patients undergoing intravenous thrombolysis, HRV serves not only as a robust biomarker for predicting 3-month clinical outcomes, but the ANS state it represents should also be considered a potential therapeutic target for improving long-term prognosis. These results establish a theoretical basis for the development of integrated treatment strategies focused on neuro-cardiac interactions.

SDNN, a time-domain measure of HRV represents the overall magnitude of HRV and is widely employed to evaluate the integrated activity of the sympathetic and parasympathetic nervous systems. In short-term resting recordings, the predominant source of HRV fluctuation is parasympathetically mediated respiratory sinus arrhythmia (RSA), especially under controlled slow-paced breathing protocols (21). Extended recordings, such as those over 24 h, encompass cardiac responses to a variety of environmental stimuli, including variations in workload, anticipatory central nervous system activity (e.g., classical conditioning), and circadian rhythms like sleep–wake cycles. Relative to shorter measurement periods (e.g., during biofeedback sessions), 24-h SDNN assessments offer superior accuracy (22). SDNN reflects the integrated contributions of both the sympathetic (SNS) and parasympathetic (PNS) nervous systems, and exhibits strong correlations with ultra-low frequency (ULF), very-low frequency (VLF), and low-frequency (LF) band power, in addition to total power. Owing to these properties, SDNN is widely regarded as the gold standard for cardiovascular risk stratification in 24-h long-term monitoring (23, 24). In clinical interpretation, SDNN and RMSSD are considered functionally complementary indices: a pronounced reduction in SDNN generally indicates sympathetic overactivation and a broad dysregulation of autonomic control, while a decline in RMSSD more specifically reflects impaired vagal activity, that is, compromised parasympathetic modulation of heart rate (15). In this investigation, the mean SDNN was significantly reduced in the unfavorable outcome group (80.58 ms) compared with the favorable outcome group (101.98 ms), approaching threshold levels linked to elevated cardiovascular risk in prior reports. This observation suggests that patients with poor outcomes may exhibit a state of systemic autonomic imbalance, marked by concurrent sympathetic hyperactivity and inadequate vagal tone. Concurrently, the specific reductions in both RMSSD and HF power (median RMSSD: 20.31 ms and median HF: 85.75 ms^2^ in the unfavorable outcome group versus 25.76 ms and 138.89 ms^2^ in the favorable outcome group) further underscore the critical role of vagal function in determining clinical prognosis. As a principal cardioprotective pathway, diminished vagal activity may directly impair cardiac electrical stability, inflammatory regulation, and endogenous repair mechanisms. Notably, both LF and VLF power were consistently lower in the unfavorable outcome group, whereas the LF/HF ratio exhibited no significant intergroup difference. This pattern supports a pathophysiological model of “generalized neurohumoral regulatory impairment” rather than one of simple “relative sympathetic overactivity,” thereby aligning with and reinforcing the holistic physiological picture reflected by SDNN.

In summary, the provision of detailed between-group descriptive data in this study effectively links the physiological interpretation of HRV parameters, particularly SDNN, to tangible patient outcomes. These findings not only corroborate the utility of SDNN as a biomarker for cardiovascular risk stratification but also underscore the critical role of vagal function in stroke recovery, thereby identifying a potential therapeutic target and establishing an evaluative framework for future individualized autonomic nervous system interventions. SDNN demonstrates predictive value for both morbidity and mortality, a prognostic significance that has been further elucidated in prior research focusing on the early phase of AIS. Tobaldini et al. (25) demonstrated that diminished parasympathetic tone upon admission correlates with worse neurological outcomes during the very early phase of acute AIS. Extending these observations, our analysis reveals that low SDNN levels independently predict unfavorable outcomes in AIS patients after IVT, even after adjustment for multiple potential confounders. Investigations into the circadian variation of HRV in AIS patients remain scarce. Under physiological conditions, the autonomic nervous system displays a distinct circadian rhythm, marked by nocturnal elevation of parasympathetic activity and a concomitant reduction in heart rate (26). Following stroke, the circadian rhythm is frequently impaired, characterized by a relative nocturnal sympathetic predominance and a marked attenuation or complete loss of normal HRV rhythmicity. Such dysregulation may increase the risk of cardiovascular events during the acute phase and hinder neurological recovery by adversely influencing cerebral perfusion and inflammatory processes, ultimately contributing to poorer clinical outcomes. In contrast to single-time-point assessments, 24-h ambulatory monitoring offers a more comprehensive evaluation of autonomic regulatory capacity and potential for recovery, thereby yielding prognostically more meaningful information. However, systematic characterization of 24-h dynamic autonomic rhythms post-stroke, particularly in the setting of acute therapeutic interventions, remains lacking. Evidence is limited regarding the independent prognostic value of circadian rhythm parameters for long-term outcomes, and the dynamic interplay between these rhythms and stroke location, etiology, and recovery phases is poorly elucidated. Our analysis of HRV during wakefulness and sleep periods suggests that reduced SDNN during wakefulness may reflect an exhaustion of autonomic reserve resulting from sustained exposure to neurohumoral factors such as cortisol and catecholamines (27), a finding consistent with prior studies examining sleep–wake HRV differences in stroke cohorts. Aftyka et al. (7) systematically reviewed that a general decline in 24-h HRV and an attenuation of circadian rhythmicity frequently occur following stroke and correlate with adverse outcomes. Nevertheless, a significant research gap persists regarding the specific and systematic comparison of wake and sleep HRV patterns in patients undergoing intravenous thrombolysis for acute ischemic stroke, as well as the investigation of their association with long-term functional outcomes. Our study not only corroborates the prevalent disruption of circadian autonomic control after stroke but, more critically, identifies reduced wakefulness SDNN as an independent predictor of long-term prognosis in the specific setting of thrombolysis. These findings provide novel mechanistic insights into how brain-heart axis interactions affect stroke recovery and imply that monitoring and modulating autonomic rhythms could serve as a potential therapeutic target to improve outcomes in this patient population.

SDNN is established as a significant independent prognostic indicator after stroke, and HRV is widely accepted as a valid biomarker for functional recovery post-stroke. Further studies indicate that ischemic stroke can induce sympathetic overactivation, promoting the infiltration of monocyte-derived macrophages into the heart and exacerbating cardiac dysfunction, thereby establishing a deleterious “brain–heart axis” vicious cycle (6). Within this mechanistic framework, vagus nerve stimulation (VNS) has emerged as a neuromodulatory intervention with demonstrated neuroprotective potential. Through multiple pathways-including anti-inflammatory, antioxidant, and anti-apoptotic mechanisms (7)—VNS has been shown to promote motor recovery in animal models of ischemic cortical injury (28) as well as in blinded randomized pilot studies of patients with chronic upper limb impairment (29). Comparative analysis in this study revealed that patients with an unfavorable prognosis exhibited significantly lower SDNN values compared to those with a favorable prognosis. This observation not only reinforces the pivotal role of cardiac autonomic dysfunction in disease progression but also indicates that individuals with substantially reduced SDNN may represent the subgroup most likely to benefit from neuromodulatory interventions, including heart rate variability biofeedback and vagus nerve stimulation. These findings prompt a key scientific question: can augmenting SDNN levels lead to improved clinical outcomes in stroke patients? Consequently, future investigations should prioritize the development of intervention strategies aimed at modulating SDNN, such as autonomic regulation training and biofeedback methodologies, thereby advancing individualized, HRV-guided neuromodulation therapies. Such an approach holds promise for establishing novel clinical pathways to enhance long-term prognosis in stroke survivors.

This study is subject to several limitations. First, although all HRV measurements were obtained within 7 days of admission, the exact timing of assessment differed across patients, potentially introducing time-dependent variability. Second, reliance on a single assessment rather than continuous monitoring precludes the detection of dynamic fluctuations in autonomic nervous activity. Future investigations should adopt longitudinal designs with serial measurements to more accurately delineate its temporal evolution. Third, as a single-center, retrospective observational study, the generalizability of the findings is inherently limited, and causal inferences cannot be firmly established. Moreover, we cannot entirely exclude the potential impact of unmeasured confounders—such as subtle complications, medication adherence, or psychosocial factors—or information bias resulting from incomplete or inconsistent clinical documentation. Additionally, given that all participants received intravenous thrombolysis and those with cardioembolic stroke were excluded, the conclusions may not be directly applicable to stroke patients who do not receive reperfusion therapy or those with concomitant cardiac disease. These limitations warrant further investigation through prospective, multicenter, large-scale cohort studies for robust validation.

## 5 Conclusion

This investigation establishes that, among patients with acute ischemic stroke treated with intravenous thrombolysis, early-phase (within 7 days post-onset) autonomic nervous system function exerts a sustained and independent influence on 90-day functional outcomes. Specifically, reduced standard deviation of normal-to-normal intervals (SDNN)—a time-domain measure of heart rate variability—was identified as an independent risk factor for an unfavorable prognosis. These observations suggest that SDNN could function as a practical biomarker for the early identification of high-risk individuals and offer new clinical evidence underscoring the significance of the brain–heart axis in stroke recovery. Collectively, these results imply that the assessment and modulation of autonomic function may constitute a novel strategic direction and a potential therapeutic target for enhancing long-term rehabilitation in this patient population.

## Data Availability

The raw data supporting the conclusions of this article will be made available by the authors, without undue reservation.
